# Deciphering the molecular nexus of BTG2 in periodontitis and diabetic kidney disease

**DOI:** 10.1186/s12920-024-01915-6

**Published:** 2024-06-03

**Authors:** Binhui Pan, Yangyang Teng, Renban Wang, Dan Chen, Hui Chen

**Affiliations:** 1 Department of Nephrology, Wenzhou Central Hospital, Wenzhou, Zhejiang Province China; 2 Department of Gastroenterology, The Second Affiliated Hospital, Yuying Children’s Hospital of Wenzhou Medical University, Wenzhou, Zhejiang Province China

**Keywords:** Diabetic kidney disease, Periodontitis, Bioinformatics analysis, EMT, Autophagy

## Abstract

**Objective:**

To investigate the role of *BTG2* in periodontitis and diabetic kidney disease (DKD) and its potential underlying mechanism.

**Methods:**

Gene expression data for periodontitis and DKD were acquired from the Gene Expression Omnibus (GEO) database. Differential expression analysis identified co-expressed genes between these conditions. The Nephroseq V5 online nephropathy database validated the role of these genes in DKD. Pearson correlation analysis identified genes associated with our target gene. We employed Gene Set Enrichment Analysis (GSEA) and Protein-Protein Interaction (PPI) networks to elucidate potential mechanisms. Expression levels of BTG2 mRNA were examined using quantitative polymerase Chain Reaction (qPCR) and immunofluorescence assays. Western blotting quantified proteins involved in epithelial-to-mesenchymal transition (EMT), apoptosis, mTORC1 signaling, and autophagy. Additionally, wound healing and flow cytometric apoptosis assays evaluated podocyte migration and apoptosis, respectively.

**Results:**

Analysis of GEO database data revealed BTG2 as a commonly differentially expressed gene in both DKD and periodontitis. BTG2 expression was reduced in DKD compared to normal conditions and correlated with proteinuria. GSEA indicated enrichment of BTG2 in the EMT and mTORC1 signaling pathways. The PPI network highlighted BTG2’s relevance to S100A9, S100A12, and FPR1. Immunofluorescence assays demonstrated significantly lower BTG2 expression in podocytes under high glucose (HG) conditions. Reduced BTG2 expression in HG-treated podocytes led to increased levels of EMT markers (α-SMA, vimentin) and the apoptotic protein Bim, alongside a decrease in nephrin. Lower BTG2 levels were associated with increased podocyte mobility and apoptosis, as well as elevated RPS6KB1 and mTOR levels, but reduced autophagy marker LC3.

**Conclusion:**

Our findings suggest that BTG2 is a crucial intermediary gene linking DKD and periodontitis. Modulating autophagy via inhibition of the mTORC1 signaling pathway, and consequently suppressing EMT, may be pivotal in the interplay between periodontitis and DKD.

## Introduction

Diabetic Kidney Disease (DKD), a prevalent complication of diabetic microangiopathy, stands as a primary cause of Chronic Kidney Disease (CKD). Notably, about 40% of individuals with type 2 diabetes and 30% of those with type 1 diabetes eventually develop kidney-related complications [1, 2]. Many patients progress to end-stage renal disease (ESRD) by the time of diagnosis. Therefore, elucidating the underlying mechanisms of DKD is crucial for early detection and intervention, significantly impacting the disease’s progression.

Periodontitis used to be considered to primarily affect oral and dental health, often resulting in tooth loss. Recently years, periodontitis has been linked to a range of systemic diseases [3–7]. Notably, in a 22-year follow-up study of type 2 diabetes patients conducted by Shultis et al. in 2007, the incidence of proteinuria and ESRD was found to increase with the severity of periodontitis. Patients with moderate to severe periodontitis, or those who were edentulous, exhibited a 2.3 to 4.9-fold higher incidence of ESRD compared to those with no or mild periodontitis [8]. Further studies have established a connection between oral pathogenic bacteria and DKD [9, 10].

However, the mechanisms linking periodontitis and DKD remain largely unexplored. In this study, we employed bioinformatics techniques and cellular functional assays to identify key interacting genes between periodontitis and DKD. The results indicated that BTG2 was the essential crosstalk gene between these two diseases. *BTG2*, part of the BTG/Tob family, regulates the cell cycle and various cellular activities [11]. As the first protein identified in BTG/Tob family, *BTG2* possesses the ability to promote programmed cell death or survival [12]. Initially studied as an antioncogene [13–16], recent research has linked *BTG2* to non-tumor conditions, including retinal microvascular endothelial cell proliferation [17], impaired blood pressure control and proteinuria [18], suggesting its potential importance in DKD. In our study, we demonstrated the decline of BTG2 in DKD and investigated the potential underlying cellular and molecular mechanisms, aiming to provide novel insights for the prevention and treatment of DKD.

## Methods

### Datasets download

Gene expression datasets for periodontitis and diabetic kidney disease (DKD) were obtained from the Gene Expression Omnibus (GEO) database (https://www.ncbi.nlm.nih.gov/geo/*).* The GSE16134 dataset, based on GPL570-55599, included 310 gingival papillae samples from 120 subjects undergoing periodontal surgery, comprising 241 “diseased” and 69 “healthy” samples. GSE10334, also based on GPL570-55599, contained 247 gingival papillae samples from 90 periodontitis patients, with 183 “diseased” and 64 “healthy” samples. For DKD, the GSE96804 dataset was based on the GPL17586 platform, including 61 samples (41 from DKD patient glomeruli and 20 from normal glomeruli from tumor nephrectomy).

### Mutual differentially expressed genes identification and correlation analysis

Differential expression analysis was set with a threshold of log2 Fold Change (FC) > 1 or <-1 and an adjusted P-value < 0.05, using the “limma” package in R software. The Nephroseq V5 online nephropathy database (https://www.nephroseq.org/*)* was used for analyzing related gene expression between DKD and normal conditions, and for clinical correlation analysis of differentially expressed genes (DEGs). DEG correlation analysis was conducted on the DKD dataset GSE96804. A correlation coefficient threshold of > 0.5 or < -0.5 and a P-value < 0.05 were used for Pearson correlation analysis. Differential analysis employed the limma package in R software with log2 FC thresholds of > 1.5 or <-1.5 and an adjusted P-value < 0.05.

### Pathway enrichment analysis and protein-protein interaction networks

DEGs between high and low expression groups, based on the target DEG expression level in GSE96804, were identified. Gene Set Enrichment Analysis (GSEA) was carried out through gene sets h.all.v7.1.symbols.gmt and c2.cp.kegg.v7.1.symbols.gmt, respectively. The related genes were screened in the STRING database, and the Protein-Protein Interaction (PPI) networks were constructed using software Cytoscape, and core gene groups were filtered by Molecular Complex Detection (MCODE).

### Antibodise and cell culture

Mouse kidney foot cells (MPC5) were purchased from Shanghai iCell Bioscience Inc (Shanghai, China) and were cultured in dulbecco’s modified eagle medium (DMEM) supplemented with 10% high quality fetal bovine serum in the humidified incubator at 37℃ 5% CO_2_. Rabbit anti-*BTG2* antibody (bs-0031R) was purchased from Beijing Bioss Biotechnology Co., Ltd while α-SMA, vimentin, Bim, pMTOR, MTOR, RPS6KB1, LC3 antibodies and nephrin, pRPS6KB1 antibodies were purchased from Wuhan Proteintech Group, Inc and Jiangsu Affinity Biosciences Ltd, respectively. Detailed procedures are described in the Supplementary Materials.

### Gene knockdown/overexpression cell lines establishment

Lentivirus-packaged BTG2-targeting siRNA was used to BTG2 knockdown, while lentivirus-packaged BTG2 plasmid was used to BTG2 overexpression. MPC5 cells were plated (24-well plate) and cultured in DMEM complete medium containing 10% FBS for 24 h to ensure a cell confluence of 20–40% and cells were in good condition. Prior to infection, we diluted the infection enhancer solution P in complete culture medium at a ratio of 24:1 and removed the original cell culture medium. After washing the cells with phosphate buffered saline (PBS), we added the medium containing the infection enhancer solution and diluted the virus to a titer of 1 × 10^8^ TU/mL by complete culture medium. Calculating the needed volume of virus according to the MOI value (MOI × number of cells / virus titer), we added 5µL of virus solution to each well for infection. After 16 h of infection, we replaced the medium with complete culture medium and cultured for an additional 48 h. Approximately 72 h post-viral infection, infection efficiency (cell growth reached 90-100%) was observed. Detailed procedures are described in the Supplementary Materials.

### Optimal time point selection for BTG2 downregulation

Quantitative polymerase chain reaction (qPCR) was used to detect the expression of BTG2 in podocytes of each group. Podocytes were planted in six-well plates and divided into normal group (Control group) and HG (30mmol/L) stimulated groups for 12 h, 24 h, 36 h and 48 h. We added 1 ml Trizol to the cells, followed by centrifuge. Then we added 200µL chloroform to the Trizol solution and then centrifuged. Added an equal volume of isopropanol (300–400µL), mixed, and held at room temperature for 10 min. After centrifuge we added 1000µL of 75% ethanol (equal volume to RNAiso Plus), mixed, and centrifuged. Washed and added 20µL DEPC to dissolve RNA, we took 2µL for RNA concentration measurement on NanoDrop. The remaining RNA was dissolved in 20µL DEPC water, then the dissolved RNA was reverse transcribed into cDNA. According to the final measurement, the optimal time point for downregulation of BTG2 stimulated by HG was selected. Detailed procedures are described in the Supplementary Materials.

### Immunofluorescence assays

First, we prepared a single-cell suspension. We preparing a six-well plate, seeded a certain number (approximately 5 × 10^5^) of cells, and cultured for 24 h or longer until the cells reach confluence of 70-80%. Then we washed each well three times with PBS, fixed with 4% pre-chilled paraformaldehyde. After discarding PBS, we blocked it with approximately 700µL of 5% BSA solution at room temperature for 1 h. Sequentially we added diluted rabbit anti-BTG2 antibody (bs-0031R, Bioss antibodies) and then incubated overnight at 4 °C. Next we recovered the primary antibody, added diluted fluorescently labeled secondary anti-rabbit antibody (4413 S, Cell Signaling Technology) and incubated at 37 °C in a water bath for 1.5 h. Finally, we discarded the secondary antibody, washed with PBS, and sealed the coverslip with mounting medium containing DAPI to quench fluorescence. Under a fluorescence microscope in the correct orientation, pictures were taken. Immunofluorescence assay verified *BTG2* expression differences under HG stimulation compared to normal conditions. Detailed procedures are described in the Supplementary Materials.

### Western Blotting (WB)

WB detected typical proteins related to epithelial-to-mesenchymal transition (EMT) (nephrin, α-SMA, and vimentin), the apoptotic protein Bim, ribosomal protein S6 kinase B1 (RPS6KB1), mammalian target of rapamycin (mTOR), and autophagy protein LC3, with β-actin as an internal reference control. In experiment, after the culture solution was discarded and cleaned twice with PBS, 100ul cell lysate was added to each well. After cell lysate was disposed, the protein sample was obtained through cracking and centrifuge, followed by albuminous degeneration. After cleaning the assembled glass plate, we prepared the separation and stacking gel according to the SDS-PAGE gel rapid preparation kit instructions. Next we put the prepared gel into the electrophoresis tank, added the electrophoresis solution and then loading. After the loading was finished, the electrophoresis solution was added for protein electrophoresis. Sequentially, a transmembrane sandwich was made and sealed with a protein-free sealing solution. The membrane was cut according to the required molecular weight, and then incubated with first and second antibody successively, and finally exposed and developed. Detailed procedures are described in the Supplementary Materials.

### Cell scratch assay

Cell scratch assay mimics the cells migration during healing in the body, which includes creating a “wound” in a monolayer of cells, capturing images at the beginning and periodically during the procudure of cell migration to close the wound, and finally comparing images for cell migration rates determination. We first used a marker pen to draw horizontal lines evenly on the back of the six-well plate, with approximately 0.5–1 cm apart, crossing through each well. Each well should have at least 5 lines. Then, we added approximately 5 × 10^5^ cells into each well. The next day, we utilized a pipette tip to make vertical scratches perpendicular to the horizontal lines on the back of the plate. After washing the cells with PBS three times, we added serum-free culture medium. We placed the plate in a 37 °C, 5% CO_2_ cell culture incubator, stimulating with HG. Finally, we sampled and took photographs at 0 and 24 h. Detailed procedures are described in the Supplementary Materials.

The scratch healing rate =(0 h scratch area − 24 h scratch area)/0 h scratch area ×100%.

### Flow cytometry

Flow cytometry further verified the effect of *BTG2* on podocyte apoptosis. Treating the cells with HG for 24 h, we washed the cells twice with PBS, then resuspended the cells at a concentration of 1 × 10^6^ cells/ml in 1× binding buffer. We then transferred 100 µl of the solution (1 × 10^5^ cells) to a 5 ml culture tube, and added 5µl of FITC Annexin V (51-65874X, BD Pharmingen) and 5µl of propidium iodide (51-66211E, BD Pharmingen). And 10X Annexin V binding buffer (51-66121E, BD Pharmingen) was diluted into distilled water to make 1X Annexin V binding buffer working liquid. After eddying the cells and incubating in the dark at room temperature (25 °C) for 15 min, 400 µl of 1X Annexin V binding buffer was added to each tube and analyzed using a flow cytometer within 1 h. The cross gate was set according to the undyed tube and the single dyed tube, so as to conduct the subsequent data analysis. Detailed procedures are described in the Supplementary Materials.

### Statistical analysis

Data from GEO database were processed using R software. Experimental data were statistically analyzed using GraphPad and Microsoft Excel. Pearson correlation analysis in R software was conducted using the “cor.test”, with a correlation coefficient threshold of > 0.5 or < -0.5, while pathway enrichment analysis was conducted using “clusterProfiler”. And differential expression analysis was carried out via the “limma” package.One-way analysis of variance was conducted for multiple group comparisons. Bonferroni correction, the final value of which was false discovery rate (FDR), was conducted for multiple hypotheses test and correction. A p value less than 0.05 was considered statistically significant.

## Results

### Mutual DEGs analysis in periodontitis and DKD

**Fig. 1:**
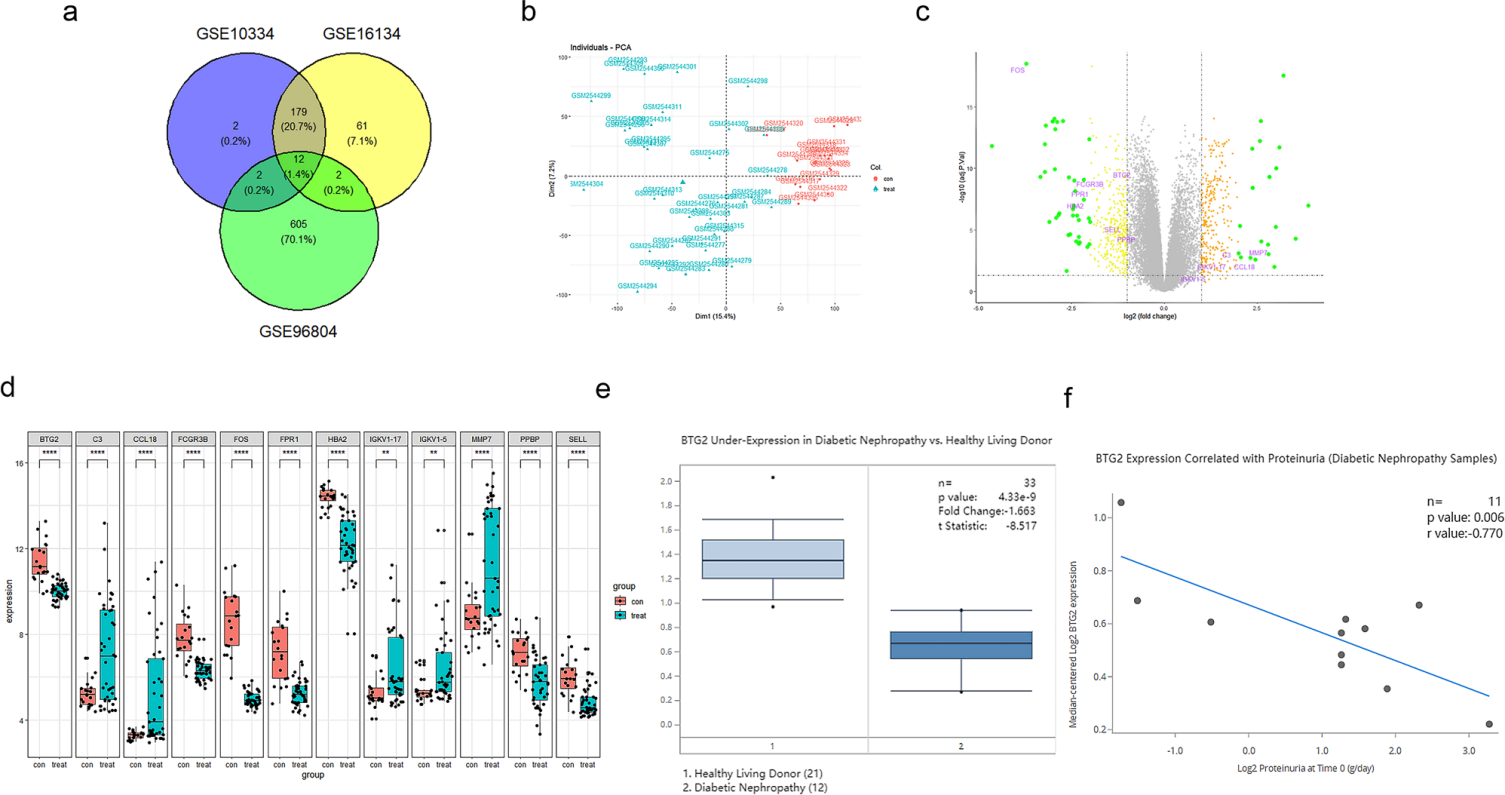
Mutual DEGs between periodontitis and DKD (**a**) Venn diagram of mutual DEGs between periodontitis (GSE16134, GSE10334) and DKD from GEO (GSE96804) database. There were a total of 12 co-expressed DEGs screened. (**b**) PCA analysis of DKD. Clustered samples indicated little difference between the samples. (**c**) Volcano plot of DEGs. Log2 FC > 1 or <-1, an adjusted P-value < 0.05. (**d**) Histogram of DEGs. Compared to the control, *BTG2, FPR1, FCGR3B, FOS, HBA2, SELL, PPBP* were decreased, and *C3, IGKV1-17, IGKV1-5, MMP7* and *CCL18* were increased. (**e**) BTG2 expression in diabetic kidney and healthy living donor from Nephroseq V5 online nephropathy database. Compared to the normal, *BTG2* expression in DKD was significantly decreased. (**f**) The correlation between *BTG2* expression and proteinuria. With higher *BTG2* expression level, DKD patients have less proteinuria. *P* < 0.05 has a statistical significance. **, *P* < 0.01; ***, *P* < 0.001; ****, *P* < 0.0001

We downloaded periodontitis and DKD gene expression datasets from GEO database. GSE16134 dataset based on GPL570-55599 and GSE10334 dataset based on GPL570-55599 contain 310 gingival papillae data from 120 subjects undergoing periodontal surgery (241 “diseased” and 69 “healthy”) and 247 gingival papillae data from 90 periodontitis patients (183 “diseased” and 64“healthy”), respectively. The DKD gene expression dataset (GSE96804) is based on GPL17586 platform, including 61 samples (41 from glomeruli of DKD patients and 20 from normal glomeruli of tumor nephrectomy). The differentially expressed genes in the three datasets through the differential analysis of limma package in R software were showed, and a total of 12 co-expressed DEGs were discovered (Fig. 1a). Although the sample size of DKD dataset was not large enough, principal component analysis (PCA) proved a high quality of this dataset (Fig. 1b). To show the results more vividly, we drawn a volcano plot for these DEGs (Fig. 1c) and details of DEGs including *BTG2*, *C3*, *IGKV1-17*, *SELL*, *FPR1*, *IGKV1-5*, *MMP7*, *FCGR3B*, *CCL18*, *FOS*, *PPBP* and *HBA2* were also established (Fig. 1d).

Among these DEGs, we found that the expression of *BTG2* in DKD patients was significantly lower than the control. Consistently, we employed Nephroseq V5 online nephropathy database and found a lower expression of *BTG2* in DKD than that in normal, with a statistical significance (Fig. 1e). The clinical correlation analysis also showed that there was a strong correlation between *BTG2* expression and proteinuria, while the *BTG2* expression level was higher, the proteinuria amount was less (Fig. 1f). Therefore, we have reason to believe that *BTG2* is a key interactive gene between periodontitis and DKD

### *BTG2* correlation analysis

**Fig. 2:**
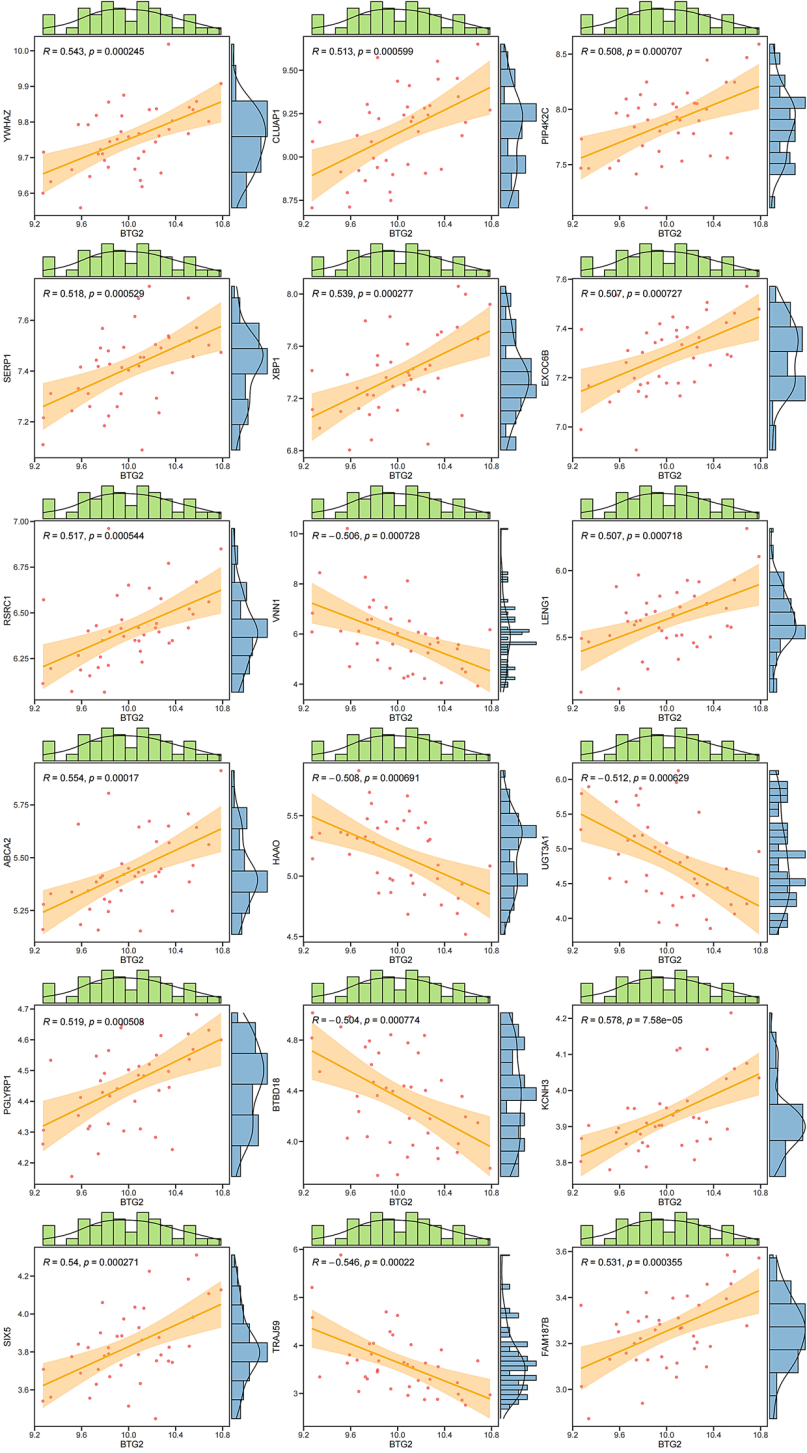
Pearson correlation analysis for BTG2 from DKD samples correlation R value stands for correlation coefficient, while positive values represent positive correlation and negative values represent negative correlation. The greater the absolute value of R, the stronger the correlation. Coefficient > 0.5 or < -0.5, *P* < 0.05

We conducted pearson correlation analysis for *BTG2* from DKD samples, and screened genes with correlation coefficient > 0.5 or < -0.5 and a p value less than 0.05. Finally, eighteen pivotal related genes were selected including “*ywhaz*”, “*Cluap1*”, “*PIP4K2C*”, “*SERP1*”, “*XBP1*”, “*EXOC6B*”, “*RSRC1*”, “*VNN1*”, “*LENG1*”, “*ABCA2*”, “*HAAO*”, “*UGT3A1*”, “*PGLYRP1*”, “*BTBD18*”, “*KCNH3*”, “*SIX5*”, “*TRAJ59*”, and “*FAM187B*” (Fig. 2).

### DEGs screened by *BTG2*

**Fig. 3:**
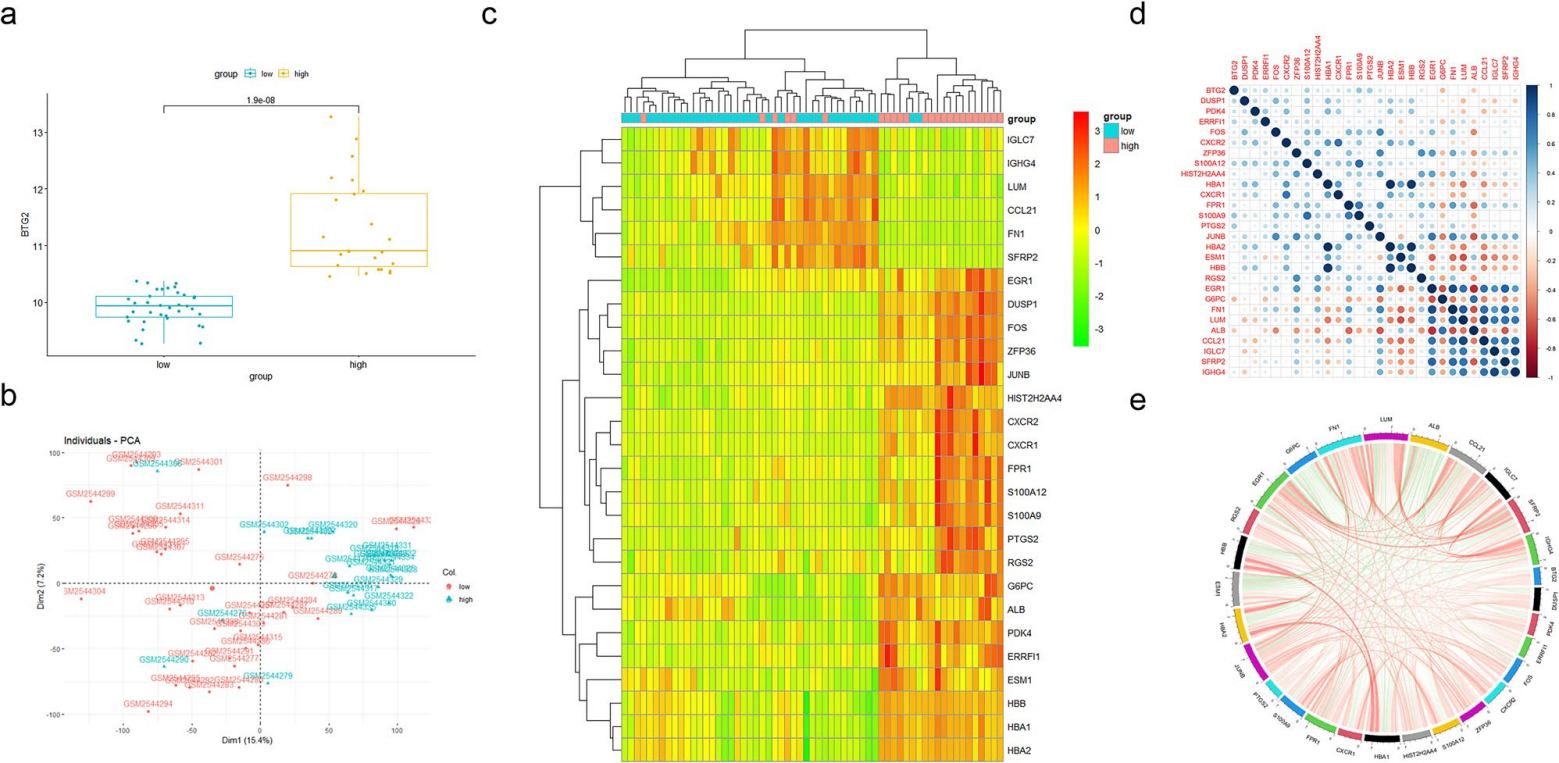
DEGs selected by *BTG2*expression level (**a**) Comparison of *BTG2* differential expression in DKD patients. Dividing DKD patients into *BTG2* higher and lower expression groups, higher *BTG2* group has significantly higher *BTG2* expression level than the lower. (**b**) PCA of *BTG2* differential expression in DKD dataset. Clustered samples indicated little difference between the samples. (**c**) Heatmap of DEGs. Demonstrated the synergistic relationship between DEGs and *BTG2* expression. (**d**) Correlation heatmap between *BTG2* and the coding genes. (**e**) Circos plot between *BTG2* and the coding genes. Log2 FC > 1.5 or <-1.5, adjust P value < 0.05

To explore the role of *BTG2* in DKD patients, we divided the DKD samples into high and low expression groups according to the *BTG2* expression level. And in the high expression group, *BTG2* expression level was significantly higher than that in the low, with a high quality of PCA (Fig. 3a and b). Thus, we set the threshold of differential analysis as log2 FC > 1.5 or <-1.5 and adjust P value < 0.05, sequentially screened out 27 coding genes, and conclusively, summarized to a heat map (Fig. 3c). Correlation analysis between *BTG2* and these coding genes were also conducted (Fig. 3d and e).

### DEGs functional analysis

**Fig. 4:**
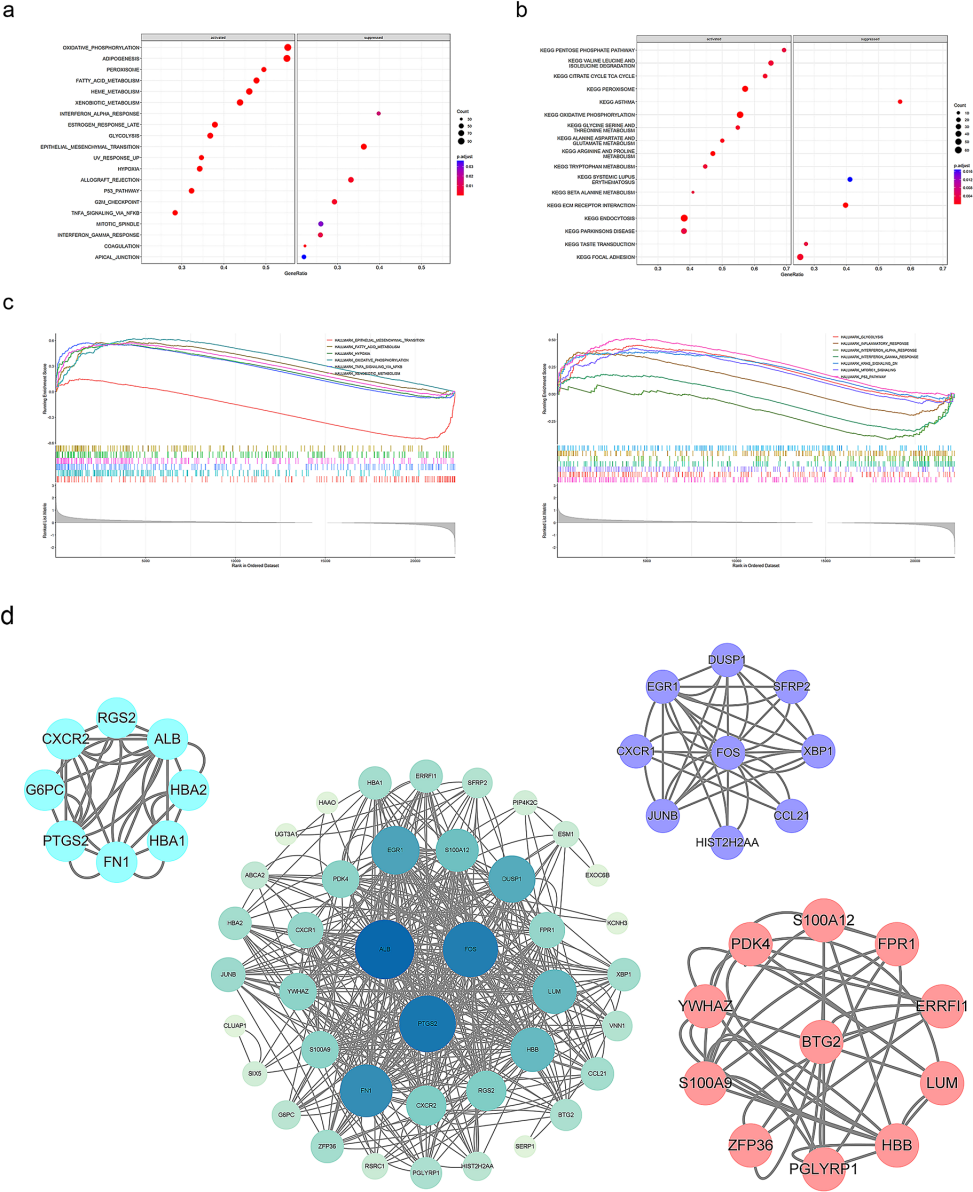
DEGs functional analysis a: Bubble diagram based on gene sets *h.all.v7.1.symbols.gmt*. b. Bubble diagram based on gene sets *c2.cp.kegg.v7.1.symbols.gmt*. Count represents bubble size and color represents adjusted *P*. GeneRatio = count / number of annotated gene. c: GSEA of BTG2 enrichment pathway. Each bar represents a gene, and the vertical coordinates represented the enrichment scores of the genes. Positive scores are regarded as gene activation, and negative scores are regarded as gene inhibition. d: PPI network constructed by cytoscape software. Parameter setting: Degree Cutoff: 2, Node Score Cutoff: 0.2, K-Core: 2, Max.Depth: 100. Cluster 1: 4.571 points, Cluster 2: 4.25 points, cluster 3: 3.6 points

To further analyze the related function of DEGs, we drawn bubble diagram utilizing the gene sets h.all.v7.1.symbols.gmt and c2.cp.kegg.v7.1.symbols.gmt (Fig. 4a/b). Since the former summarized and represented specific well-defined biological states or processes and displayed coherent expression, we further utilized this gene set for pathway enrichment analysis and finally a total of 29 related DEGs enriched pathways were screened. As the figure displayed (Fig. 4c), each bar represents a gene, and the sequence of genes was ordered according to the log2 FC of all these genes. However, only the genes enriched in special function would be displayed. The vertical coordinates represented the enrichment scores of the genes. Positive scores could be regarded as gene activation, and negative scores could be regarded as gene inhibition. The results showed that DEGs were enriched in EMT pathway and mTORC1 signal pathway, suggesting that *BTG2* might affect DKD prognosis through EMT and mTORC1 pathway.

Then, we gathered the above 18 highest correlated genes and the 27 DEGs in the STRING database, constructed PPI network by cytoscape software (Fig. 4d). Ultimately, we screened out three core gene groups by MCODE, among which the *BTG2* located in proving that *BTG2* was relevant to *S100A9*, *S100A12*, FPR1 and some other genes.

### Cellular functional assays

**Fig. 5:**
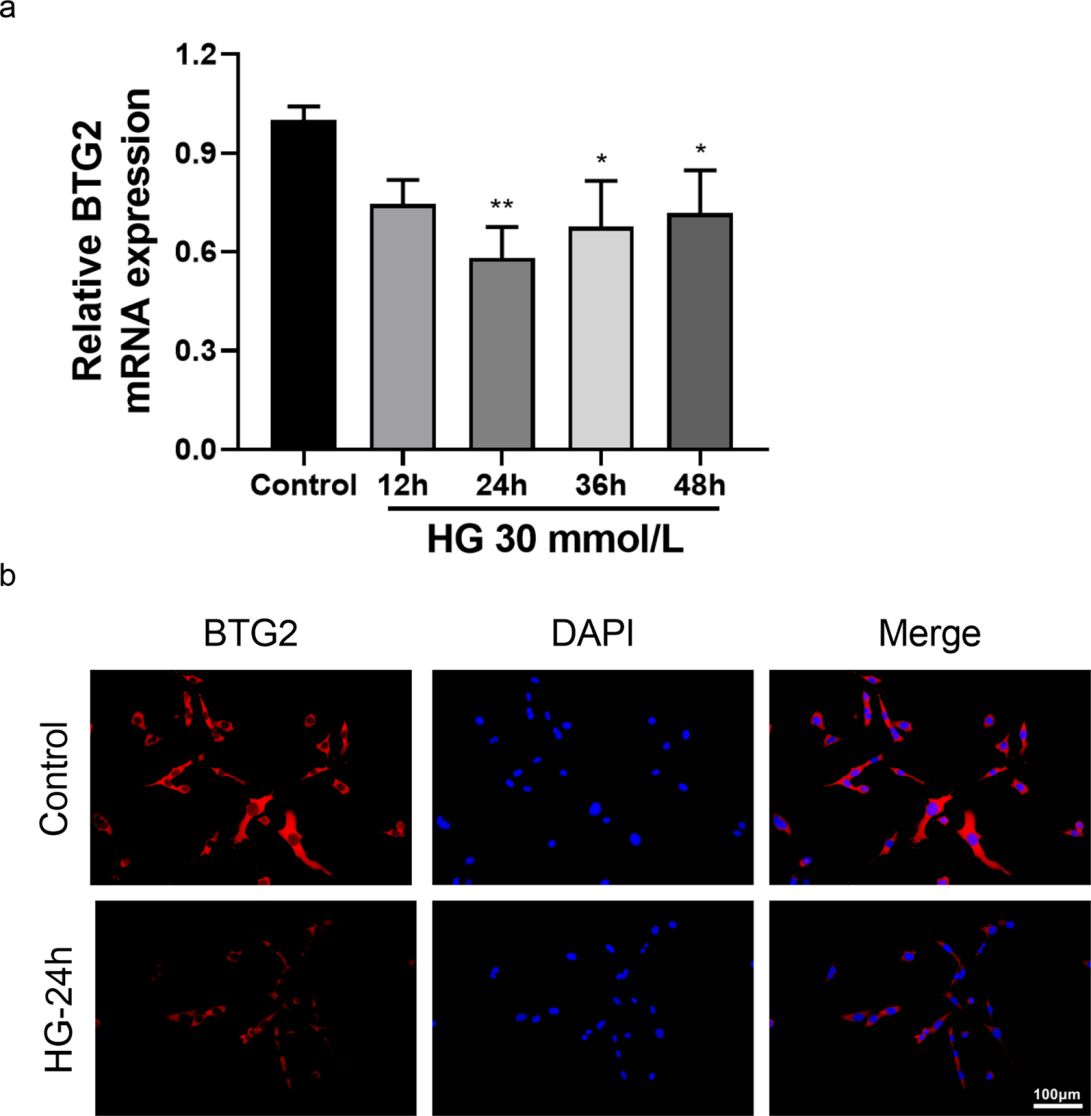
BTG2 optimal exposure time determination and expression change to HG **a**:the BTG2 mRNA expression level under different HG (30mmol/L) exposure time detected by aPCR. When exposed to HG for 24 h, *BTG2* has the lowest expression level. **b**: BTG2 expression change when exposed to HG in immunofluorescence assay. *, *P* < 0.05; **, *P* < 0.01

In order to test our hypothesis, we performed cellular functional assays. First, we used qPCR observe the *BTG2* mRNA expression level under different HG exposure time (Fig. 5a). We found that 24 h-HG exposure lead to a lowest BTG expression level, so 24 h was set as the optimal exposure time. Immunofluorescence assay showed that compared with normal podocytes, the expression level of *BTG2* in podocytes dealt with HG was significantly reduced (Fig. 5b), which was consistent with the conclusion drawn by our bioinformatics analysis.

**Fig. 6:**
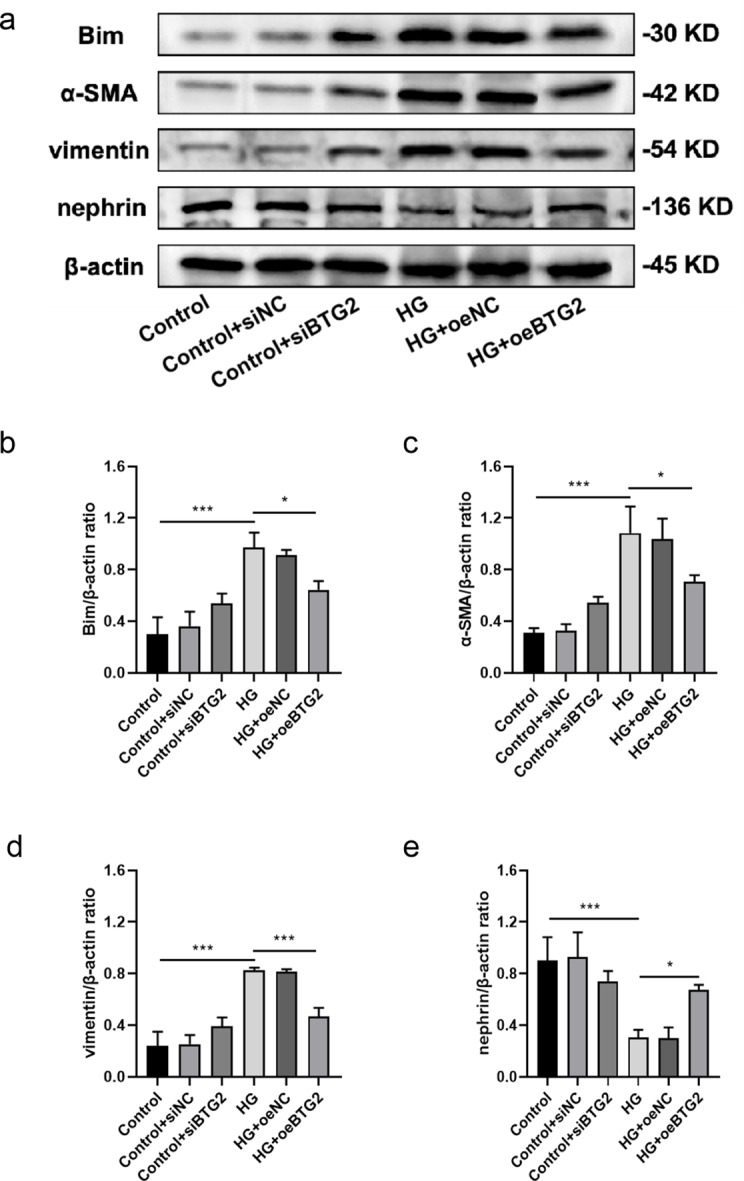
The relationship between BTG2 and EMT and apoptotic related proteins **a**: Western blot between BTG2 and nephrin, α-SMA, vimentin, Bim. **b**: Histogram of BTG2’s relation with Bim. Compared to the control, exposed to HG had a higher Bim/β-actin ratio. Compared to HG exposure, over-expressing *BTG2* under HG had a lower Bim/β-actin ratio. **c**: Histogram of BTG2’s relation with α-SMA. Compared to the control, exposed to HG had a higher α-SMA/β-actin ratio. Compared to HG exposure, over-expressing *BTG2* under HG had a lower α-SMA/β-actin ratio. **d**: Histogram of BTG2’s relation with vimentin. Compared to the control, exposed to HG had a higher vimentin/β-actin ratio. Compared to HG exposure, over-expressing *BTG2* under HG had a lower vimentin/β-actin ratio. e: Histogram of BTG2’s relation with nephrin. Compared to the control, exposed to HG had a lower nephrin/β-actin ratio. Compared to HG exposure, over-expressing *BTG2* under HG had a higher α-SMA/β-actin ratio. Internal reference control: β-actin; NC: negative control; *, *P* < 0.05; ***, *P* < 0.001

**Fig. 7:**
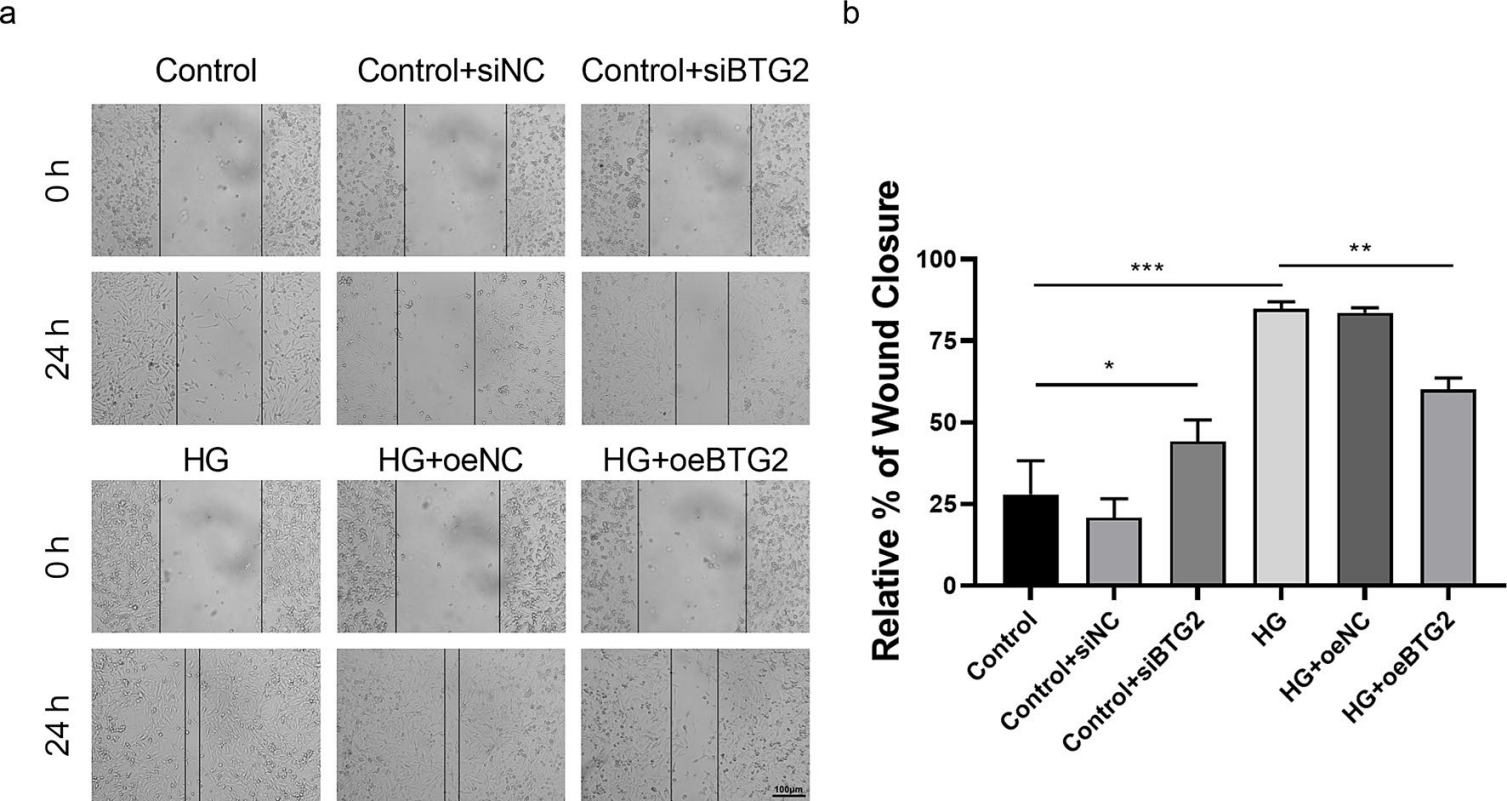
The relationship between *BTG2* expression and podocytes migration ability **a**. Wound healing assay of *BTG2* expression to cell migration ability. The wound closure rates were observed to compare cell migration ability under the condition of *BTG2* underexpression and overexpression. **b**: Relative % of wound closure. Compared to the control, under-expressing *BTG2* and exposed to HG had better wound closure. Compared to HG exposure, over-expressing *BTG2* under HG had worse wound closure. NC: negative control; *, *P* < 0.05; ***, *P* < 0.001

**Fig. 8:**
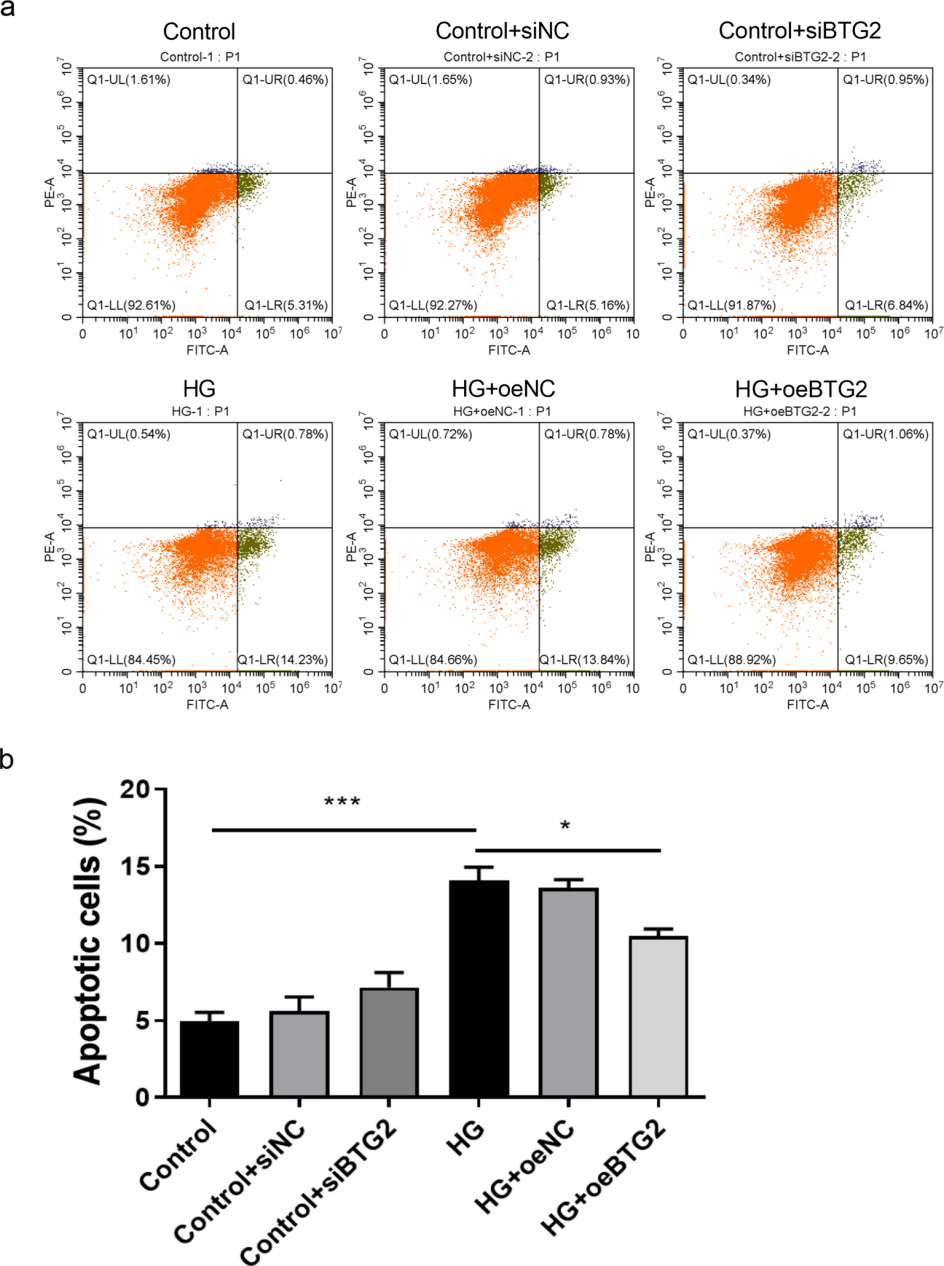
The relationship between BTG2 expression and podocyte apoptosis **a**: Flow apoptosis assay of BTG2 to cell apoptosis. **b**: Apoptosis cells (%) with BTG2 expression change. Compared to the control, exposed to HG had much more apoptotic cells. Compared to HG exposure, over-expressing *BTG2* under HG had less apoptotic cells. NC, negative control; si, under-express; oe, over-express. *, *P* < 0.05; ***, *P* < 0.001

Sequentially, the expression levels of EMT-related proteins (nephrin, α-SMA and vimentin) and apoptotic protein Bim were also detected via WB method (Fig. 6). According to the existing literature, α-SMA and vimentin are two cytoskeletal proteins, in which α-SMA is a marker of smooth muscle cells, while vimentin is an intermediate product of renal tubular epithelial cells during their transformation into myofibroblasts [19, 20]. And nephrin is specifically expressed in the podocyte hiatus membrane, the loss of which could destroy the integrity of the glomerular filtration barrier, further incur massive proteinuria [21]. Protein Bim, as one of the Bcl-2 family proteins, could promote cell apoptosis. In this experiments, we discovered the decrease of *BTG2* stimulated by HG significantly corresponded to the decrease of nephrin and the increase of α-SMA, vimentin and Bim. And overexpression of *BTG2* could partially reverse this phenomenon, suggesting that *BTG2* could inhibit the EMT and reduce the podocytes apoptosis. Cell scratch assay is a method for cell migration assessment. The target cells were placed in a culture medium with scratches and then observed how the cells repaired these injuries and migrated to adjacent areas. Through statistical analysis of the percentage of Cell scratch distance between scratches, it is possible to visualize cells migration ability. The results showed that compared with normal podocytes, the migration ability of podocytes was significantly improved after inhibiting *BTG2* or exposure to HG (Fig. 7), while gene overexpression could retard the migration, suggesting a control of *BTG2* to EMT in a way. Notably, EMT refers to a stronger migration ability. Flow cytometry was also conducted to probe the influence of *BTG2* on podocyte apoptosis (Fig. 8). We employed flow cytometer measuring podocutes fluorescence intensity and staining characteristics treated with HG and further analyzed statistically. We found when the expression of *BTG2* decreased, the percentage of apoptotic cells significantly increased, and this manifestation was also alleviated by *BTG2* overexpression.

**Fig. 9:**
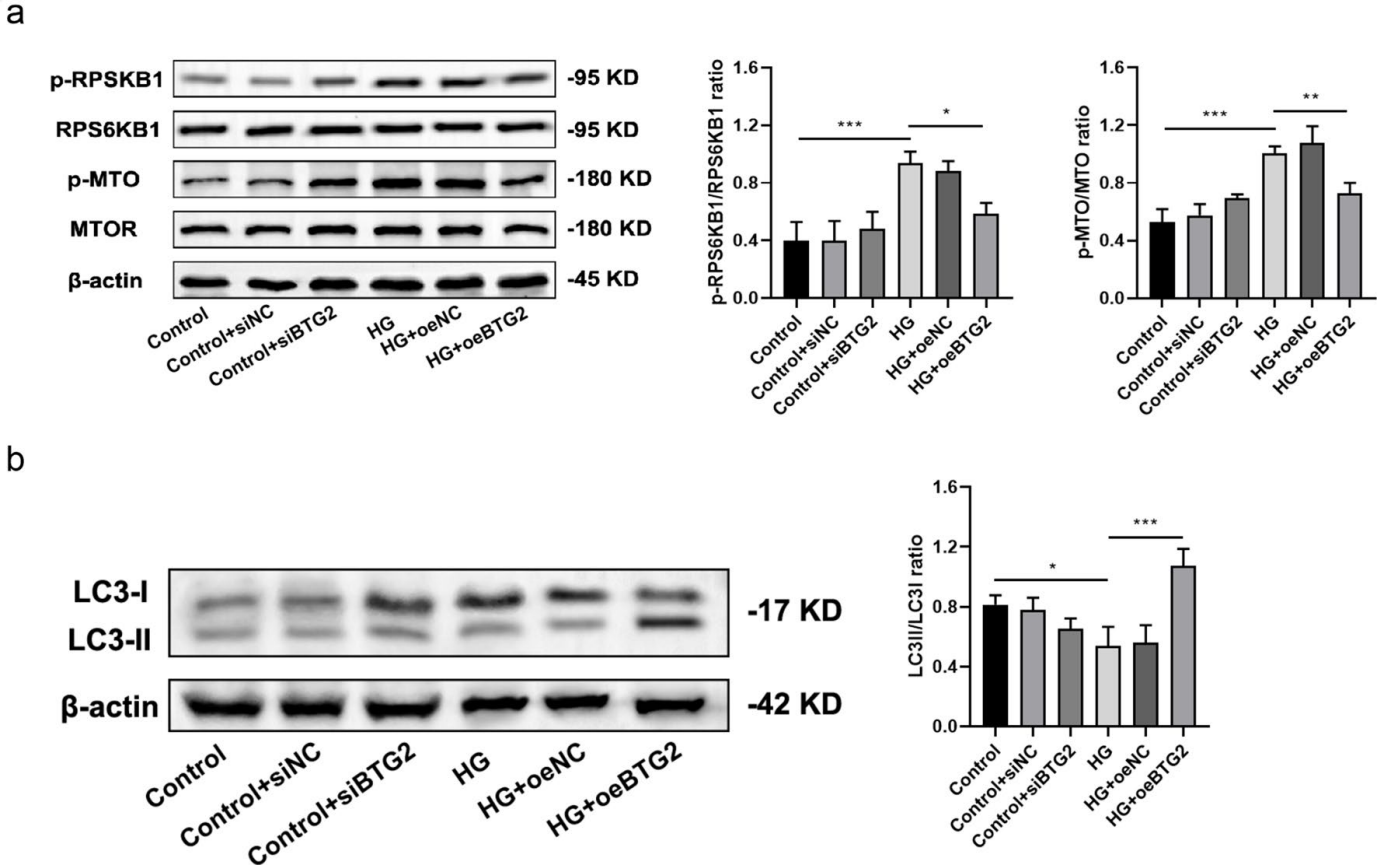
The relationship between BTG2 and mTORC1 signaling pathway and autophagy related proteins **a**: Western blot of BTG2 to RPS6KB1 and mTORC1. Compared to the control, exposed to HG had higher p-RPS6KB1/RPS6KB1 and p-MTORC1/MTORC1 ratios. Compared to HG exposure, over-expressing BTG2 under HG had lower p-RPS6KB1/RPS6KB1 and p-MTORC1/MTORC1 ratios. **b**: Western blot of BTG2 to autophagy protein LC3. Compared to the control, exposed to HG had a lower LC3II/LC3I ratio. Compared to HG exposure, over-expressing BTG2 under HG had a higher LC3II/LC3I ratio. Internal reference control: β-actin; NC: negative control; *, *P* < 0.05; **, *P* < 0.01; ***, *P* < 0.001

Finally, we examined the effects of *BTG2* expression on mTORC1 signaling pathway and autophagy using WB (Fig. 9). It was found that compared with the normal, exposure to HG gave rise to increases in pRPS6KB1/RPS6KB1 ratio and mTOR/TOR ratio, while a decrease in autophagy protein LC3 II/LC3 I ratio was detected. And these were partially reversed when *BTG2* was overexpressed, suggesting *BTG2*’s possible promotion of autophagy via mTORC1 signaling pathway inhibition.

## Discussion

Diabetic Kidney Disease (DKD) is rapidly becoming a leading cause of Chronic Kidney Disease (CKD) and its associated morbidity and mortality [22]. Extensive research has established connections between DKD and other conditions, such as periodontitis [23–25]. Notably, significant differences in the oral flora of periodontitis patients have been observed between DKD patients and diabetics without DKD [26]. The influence of oral and periodontal microflora on systemic diseases extends beyond local inflammatory reactions, involving the dissemination of oral microbial virulence through the bloodstream, oropharynx, and respiratory tract [27]. This increasing prevalence of DKD necessitates deeper investigation into its association with periodontitis.

With the advancement of bioinformatics, we supplied this method to probe the interaction between DKD and periodontitis. We downloaded and analyzed the gene expression data sets in GEO. The results showed that the gene *BTG2* was a common DEG of periodontitis and DKD. *BTG2* was underexpressed significantly in DKD than the normal and correlated with clinical prognostic indicators like albuminuria.When probing the possible pathway that *BTG2* affects on DKD, we firstly organized GSEA, a bioinformatics tool detecting the enrichment pathway of target gene, and the results indicated that EMT pathway as well as mTORC1 signaling pathway might involve in the way *BTG2* works between periodontitis and DKD. EMT refers to the biological process epithelial cells transformed into cells with mesenchymal phenotype, and the main characteristics are the reduction of the expression of cell adhesion molecules (such as E-cadherin), the transformation of cytoskeleton from keratin to vimentin, and the morphological characteristics of mesenchymal cells [28, 29]. About mTORC1 signaling pathway, pivotal protein mTORC1 is a vital regulator of EMT. In the way to EMT, mTORC1 is conducive to cell enlargement, protein synthesis, motility and invasion, while mTORC2 is necessary for epithelial to mesenchymal phenotype transition [30–32]. According to the literature published, EMT is a pivotal contributor for renal fibrosis, since about 30% of fibroblasts are derived from the tubular epithelial cells via EMT [33, 34]. Hyperglycaemia can induce EMT, and upon EMT, or other stimuli induced by high glucose, podocytes can change their epithelial cells phenotype to mesenchymal cells [35]. Finally, EMT of tubular epithelial cells and glomerular podocytes would incur tubulointerstitial fibrosis and glomerulosclerosis, which indicate a poor long-term outcomes [36].

Our study observed the changes of EMT-related and mTORC1 signaling pathway proteins in podocytes when the *BTG2* expression was reduced under the HG condition. We discovered that, compared with the normal, proteins had a significant increase, representing a EMT and mTORC1 signaling pathway promotion. Cell scratch assay showed that compared with the control, reducing the *BTG2* expression in normal podocytes could promote cell motility, when this was more apparent in the group exposed to HG. As we all know, the remarkable feature of EMT is a stronger migration ability. Thus the assay reflected the inhibition of *BTG2* to EMT from the side. Correspondingly, up-regulation *BTG2* again in HG group restraining the cell motility corroborated this suppose. This showed that *BTG2* could inhibit EMT through mTORC1 pathway.

Considering decreased mTORC1 activity can induce autophagy, we also detected changes of autophagy protein following the *BTG2* expression level. Autophagy is a significant mechanism for cellular homeostasis maintenance through potentially toxic components removement and degraded substances recycle [37]. Autophagy mediate EMT, and loss of autophagy will dramatically promote the EMT [38]. Aberrant autophagy is the main character of CKD and autophagy inducement can protect kidney from damage [39, 40]. In this study, we found when *BTG2* was inhibited, autophagy protein had a significant decrease while overexpression of *BTG2* could reverse this tendency. So we have enough reason to believe that *BTG2* could inhibit mTORC1 signaling pathway, enhance autophagy, therefore restraining EMT.

Although the related of *BTG2* in this pathway have not yet been further confirmed, but the PPI network in this study conducted demonstrated that *BTG2* was relevant to the genes *S100A9* and *S100A12*. Both belong to the S100 protein family, *S100A9* and *S100A12* are linked with systematic inflammation while sustained inflammation represents the deterioration of renal interstitial fibrosis [41–44]. Targeting the *S100A9* signaling with inhibitors could decrease inflammation, ameliorate kidney injury, improve renal function and improved long-term outcomes such as mortality reduction with decreased renal fibrosis [45]. *S100A12* was declared to show expression in renal endothelial cells [42] and was deemed as a predictor of increased CKD mortality risk in a manner that was independent of the soluble advanced glycation end-products receptor [46]. Combined with these published articles, we speculate that *BTG2* may have a correlation with *S100A9* and *S100A12* in the way it actions on the EMT pathway.

Interestingly, our cell experiments also revealed *BTG2*’s role in reducing podocyte apoptosis. This finding is intriguing, given the traditional view of EMT as being anti-apoptotic. It has been demonstrated that in many cells including renal cells, EMT and apoptosis concomitantly occurs [47–49]. Therefore, we hold the opinion that *BTG2* might independently influence apoptosis, separate from its effects on EMT, which should be warranted with further investigation.

Considering *BTG2* as the target gene that affecting DKD, regulating the upstream factors or pathways that lead to the *BTG2* decrease to increase *BTG2* expression in DKD, further affecting subsequent reactions including EMT and autophagy, could finally influence the occurrence and development of DKD. However, due to time and resource limitations, we have not proved the potential mechanism of how *BTG2* affect apoptosis yet, and we did not further detect the detailed proteins or molecules involved in the EMT pathway *BTG2* located, which would restrict the clinical development and application of drug targeted therapy. We will conduct further explorations in the future to examine the essential role of *BTG2* and detailed pathway *BTG2* located between periodontitis and DKD.

## Conclusion

In conclusion, our study used both bioinformatic analysis and cellular functional assays to testify that *BTG2* emerges as a key gene in the interaction between DKD and periodontitis, potentially up-regulating autophagy by inhibiting the mTORC1 signaling pathway, thereby inhibiting EMT.

## Data Availability

The full uncropped Gels and Blots images supporting the conclusions of this article and method details are included in the supplementary file. Periodontitis and DKD data were deposited into the Gene Expression Omnibus database under accession number GSE16134, GSE10334 and GSE96804, which are available at the following URL: https://www.ncbi.nlm.nih.gov/geo/query/acc.cgi? acc=GSE16134, https://www.ncbi.nlm.nih.gov/geo/query/acc.cgi? acc=GSE10334, https://www.ncbi.nlm.nih.gov/geo/query/acc.cgi? acc=GSE96804.

## Abbreviations

CKD: Chronic Kidney Disease
DEGs: Differentially Expressed Genes
DKD: Diabetic Kidney Disease
EMT: Epithelial-To-Mesenchymal Transition
GEO: Gene Expression Omnibus
GSEA: Gene Set Enrichment Analysis
HG: High Glucose
MCODE: Molecular Complex Detection
mTOR: Mammalian Target of Rapamyci
PBS: Phosphate Buffered Saline
PPI: Protein-Protein Interaction
qPCRF: Quantitative Polymerase Chain Reaction
RPS6KB1: Ribosomal Protein S6 Kinase B1
WB: Western Blotting
